# Supplementary material for: Envisaging the job satisfaction and turnover intention among the young workforce: Evidence from an emerging economy
Source: PLoS One. 2023 Jun 16;18(6):e0287284. doi: 10.1371/journal.pone.0287284 (PMC10275453; doi:10.1371/journal.pone.0287284)
Supplement: S1 File — (DOCX) [file pone.0287284.s002.docx]

**Supplementary Material 1.** Survey Instrument

| PC1 | The organization offers the career opportunities that are attractive to me | Murtha et al., (1998);  Kraimer et al., (2011) |
| --- | --- | --- |
| PC2 | The organization offers career opportunities that are available and of interest to me |  |
| PC3 | The organization offers many career opportunities that match my career goals |  |
| PC4 | The organization offers me equal or better career opportunities, compared to my organization counterparts around the world |  |
| PC5 | The organization offers me equal career opportunity anywhere in the world, compared to my organization counterparts |  |
| PP1 | The compensation plans of the organization reward out-standing job performance | Nyberg (2010); Heneman et al., (1988) |
| PP2 | The pay will be better when the performance is better. |  |
| PP3 | This organization recognizes productive people |  |
| PP4 | The pay raises that I receive on my job make me work harder |  |
| PP5 | High performers and low performers seem to get different pay raises |  |
| AC1 | I feel a strong sense of belonging to the organization | Humborstad & Perry (2011);  Stazyk et al., (2011) |
| AC2 | I feel that the organization has a great deal of personal meaning for me. |  |
| AC3 | I feel “emotionally attached” to the organization |  |
| AC4 | I really feel as if this organization’s problems are my own. |  |
| AC5 | I would feel very happy to spend the rest of my career with this organization |  |
| JS1 | I feel fairly well satisfied with my present job | Judge et al., (1998); Zheng et al., (2015) |
| JS2 | I feel that I find real enjoyment in my work |  |
| JS3 | Most days I feel enthusiastic about my work |  |
| JS4 | I feel basically satisfied with my work achievements in my current job |  |
| JS5 | I feel that work is a meaningful experience |  |
| JA1 | I can make my own decisions about how to schedule my work | Morgeson & Humphrey (2006);  Thompson & Prottas (2006) |
| JA2 | I can decide on the order in which things are done on the job |  |
| JA3 | It is basically my own responsibility to decide how my job gets done |  |
| JA4 | I can have a lot of say about what happens on my job |  |
| JA5 | I can decide when I take breaks |  |
| TI1 | I think a lot about leaving the organization | Mobley et al., (1978); Humborstad & Perry (2011) |
| TI2 | I think that this organization is not the best of all possible organizations to work for |  |
| TI3 | I am not very likely to stay in this organization for the next five years |  |
| TI4 | I will leave the organization as soon as possible |  |
| TI5 | I will give up this organization easily |  |
| TI6 | I am actively searching for an alternative to the organization |  |
