# Supplementary material for: Envisaging the job satisfaction and turnover intention among the young workforce: Evidence from an emerging economy
Source: PLoS One. 2023 Jun 16;18(6):e0287284. doi: 10.1371/journal.pone.0287284 (PMC10275453; doi:10.1371/journal.pone.0287284)
Supplement: Attachment — Submitted filename: Reply to Reviewers Comments.docx [file pone.0287284.s003.docx]

PONE-D-23-03002

Career Development Opportunity, Remuneration, and Organisational Commitment on Job Satisfaction and Turnover Intention: Moderating Effect of Job Autonomy

PLOS ONE

Dear Dr. Al Mamun,

Thank you for submitting your manuscript to PLOS ONE. After careful consideration, we feel that it has merit but does not fully meet PLOS ONE’s publication criteria as it currently stands. Therefore, we invite you to submit a revised version of the manuscript that addresses the points raised during the review process.

Please submit your revised manuscript by **Apr 14 2023** 11:59PM. If you will need more time than this to complete your revisions, please reply to this message or contact the journal office at [plosone@plos.org](mailto:plosone@plos.org). When you're ready to submit your revision, log on to <https://www.editorialmanager.com/pone/> and select the 'Submissions Needing Revision' folder to locate your manuscript file.

Please include the following items when submitting your revised manuscript:

- A rebuttal letter that responds to each point raised by the academic editor and reviewer(s). You should upload this letter as a separate file labeled 'Response to Reviewers'.
- A marked-up copy of your manuscript that highlights changes made to the original version. You should upload this as a separate file labeled 'Revised Manuscript with Track Changes'.
- An unmarked version of your revised paper without tracked changes. You should upload this as a separate file labeled 'Manuscript'.

If you would like to make changes to your financial disclosure, please include your updated statement in your cover letter. Guidelines for resubmitting your figure files are available below the reviewer comments at the end of this letter.

If applicable, we recommend that you deposit your laboratory protocols in [protocols.io](http://protocols.io) to enhance the reproducibility of your results. Protocols.io assigns your protocol its own identifier (DOI) so that it can be cited independently in the future. For instructions see: <https://journals.plos.org/plosone/s/submission-guidelines#loc-laboratory-protocols>. Additionally, PLOS ONE offers an option for publishing peer-reviewed Lab Protocol articles, which describe protocols hosted on [protocols.io](http://protocols.io). Read more information on sharing protocols at <https://plos.org/protocols?utm_medium=editorial-email&utm_source=authorletters&utm_campaign=protocols>.

We look forward to receiving your revised manuscript.

Kind regards,

Bo Pu, Ph.D.
Academic Editor
PLOS ONE

**Journal requirements:**

When submitting your revision, we need you to address these additional requirements.

1.  Please ensure that your manuscript meets PLOS ONE's style requirements, including

those for file naming. The PLOS ONE style templates can be found at

<https://journals.plos.org/plosone/s/file?id=wjVg/PLOSOne_formatting_sample_main_body.pdf>  and

<https://journals.plos.org/plosone/s/file?id=ba62/PLOSOne_formatting_sample_title_authors_affiliations.pdf>

**Author(s) Reply:** Thank you for your constructive comments and suggestions. Amended accordingly

2. Please include the information provided on ethical considerations and participant consent in the methods section of the manuscript.

**Author(s) Reply:** Thank you for your constructive comments and suggestions. Amended accordingly:

Prior to the commencement of the formal questionnaire, all participants signed an informed consent form, in which they were informed of the purpose of the data collection, the final destination of the data, and their right to withdraw from participation at any time. The local ethics committee (Ethical Review of Biomedical Research Involving Human Beings, China) ruled that no formal ethics approval was required because the data is anonymous, not sensitive or confidential in nature and no vulnerable or dependent groups included. At the end of the data collection, this study successfully obtained 532 valid questionnaire sets.

3. Please ensure that you include a title page within your main document. You should list all authors and all affiliations as per our author instructions and clearly indicate the corresponding author.

**Author(s) Reply:** Thank you for your constructive comments and suggestions. Amended accordingly:

Chen Xuelin^1,2^, Abdullah Al Mamun*^2^, Wan Mohd Hirwani Wan Hussain^2^, Gao Jingzu^2^, Qing Yang^2^, Sayed Samer Ali Al Shami^3^

^1^School of Business, Jishou University, 416000 Jishou City, Hunan, China.

**^2^**UKM - Graduate School of Business, Universiti Kebangsaan Malaysia, 43600, UKM Bangi, Selangor Darul Ehsan, Malaysia

**^3^**Institute of Technology Management and Entrepreneurship, Universiti Teknikal Malaysia Melaka, Melaka, Malaysia

**Address:** UKM - Graduate School of Business, Universiti Kebangsaan Malaysia, 43600, UKM Bangi, Selangor Darul Ehsan, Malaysia. Email: almamun@ukm.edu.my/mamun7793@gmail.com

4. Please include your full ethics statement in the ‘Methods’ section of your manuscript file. In your statement, please include the full name of the IRB or ethics committee who approved or waived your study, as well as whether or not you obtained informed written or verbal consent. If consent was waived for your study, please include this information in your statement as well.

**Author(s) Reply:** Thank you for your constructive comments and suggestions. Amended accordingly:

Prior to the commencement of the formal questionnaire, all participants signed an informed consent form, in which they were informed of the purpose of the data collection, the final destination of the data, and their right to withdraw from participation at any time. The local ethics committee (Ethical Review of Biomedical Research Involving Human Beings, China. Link: <http://www.gd.gov.cn/zwgk/wjk/zcfgk/content/post_2530813.html>) ruled that no formal ethics approval was required because the data is anonymous, not sensitive or confidential in nature and no vulnerable or dependent groups included. At the end of the data collection, this study successfully obtained 532 valid questionnaire sets.

**ADDITIONAL EDITOR COMMENTS:**

Dear Abdullah Al Mamun,

Thank you for submitting your manuscript, Manuscript Number PONE-D-23-03002 and entitled "Career Development Opportunity, Remuneration, and Organisational Commitment on Job Satisfaction and Turnover Intention: Moderating Effect of Job Autonomy," to the PLOS ONE.

I find the topic of career development and organization behavior to be interesting and relevant, I have decided to major revision. Looking forward your modified version.


Best Wishes,

Bo Pu, Ph.D.
Academic Editor
PLOS ONE


[Note: HTML markup is below. Please do not edit.]

Reviewers' comments:
Reviewer's Responses to Questions

**Comments to the Author**

1. Is the manuscript technically sound, and do the data support the conclusions?

The manuscript must describe a technically sound piece of scientific research with data that supports the conclusions. Experiments must have been conducted rigorously, with appropriate controls, replication, and sample sizes. The conclusions must be drawn appropriately based on the data presented.

Reviewer #1: Yes

Reviewer #2: Yes

2. Has the statistical analysis been performed appropriately and rigorously?

Reviewer #1: Yes

Reviewer #2: Yes

3. Have the authors made all data underlying the findings in their manuscript fully available?

The [PLOS Data policy](http://www.plosone.org/static/policies.action#sharing) requires authors to make all data underlying the findings described in their manuscript fully available without restriction, with rare exception (please refer to the Data Availability Statement in the manuscript PDF file). The data should be provided as part of the manuscript or its supporting information, or deposited to a public repository. For example, in addition to summary statistics, the data points behind means, medians and variance measures should be available. If there are restrictions on publicly sharing data—e.g. participant privacy or use of data from a third party—those must be specified.

Reviewer #1: Yes

Reviewer #2: No

4. Is the manuscript presented in an intelligible fashion and written in standard English?

PLOS ONE does not copyedit accepted manuscripts, so the language in submitted articles must be clear, correct, and unambiguous. Any typographical or grammatical errors should be corrected at revision, so please note any specific errors here.

Reviewer #1: Yes

Reviewer #2: Yes

5. Review Comments to the Author

Please use the space provided to explain your answers to the questions above. You may also include additional comments for the author, including concerns about dual publication, research ethics, or publication ethics. (Please upload your review as an attachment if it exceeds 20,000 characters)

**REVIEWER #1:**

**Comment 1:** 1. The introduction part is too long-winded, the author needs to be more targeted to explain the research topic, to comb out the explanation of the research gap

**Author(s) Reply:** Thank you for your constructive comments and suggestions, Prof. Amendment highlighted (added – **BLUE**; removed RED) below:

**INTRODUCTION**

~~It has become increasingly crucial for companies to enhance their work efficiency due to the intense market competition. The workforce has substantial influence on the organisational or business success, which explains the significant need for companies to optimise their workforce, particularly the younger workforce considering their high turnover rate and independent nature (Ramlah et al., 2021).~~ With the constant development of the economy and the increasing competition in the labour market, an increasing number of young employees are contemplating job-hopping or leaving their current company. The higher the turnover intention, the higher the likelihood of quitting the job (Jacobs & Roodt, 2008). Belete (2018) described turnover intention as the intention to leave the workplace. The management must have good understanding of the factors that contribute to turnover intention considering how a higher turnover rate would affect the organisational morale, reduce the sense of identity within the company (Joe et al., 2018), and yield negative financial impact since the companies have to allocate resources for recruitment and training and experience brain drain and lower productivity (Belete, 2018).

The older workforce possesses more traditional values, such as contributing to the country and supporting the family, but the younger workforce emphasises more worldly values and embraces self-development as the key purpose of working (Zhao, 2018). In general, the younger workforce demonstrates unique characteristics at work. They have different expectations at work and expect higher expectations on perceived career development opportunity (PC) and perceived pay for performance (PP) than the older workforce (Magni & Manzoni, 2020). Besides that, prior studies demonstrated lower affective organisational commitment (AC) among young employees than older employees (Singh & Gupta, 2015; Salminen & Miettinen, 2019). ~~Adding to that, the younger workforce highly values job autonomy (JA) at the workplace. Morgeson and Humphrey (2006) defined JA as the extent of freedom and independence allowed for employees to arrange their work schedule, make decisions, and select the method of performing tasks at the workplace. The younger workforce is more independent in nature (Ramlah et al., 2021) and may be less loyal towards the company (Singh & Gupta, 2015). They are not willing to obey orders and instructions, as they prefer to work freely and independently (Lee et al., 2017). Moreover, the younger workforce emphasises how they feel, which makes them highly value job satisfaction (JS) at the workplace (Yi et al., 2010; Zhao, 2018).~~ In short, unlike the older workforce, the younger workforce has higher expectations on PC and PP but lower expectations on AC compared with older workforce ~~and display higher concern for JA and JS.~~

Prior studies examined the influence of these unique attributes of young workforce on turnover intention (TI) (Lee et al., 2017; Belete, 2018; Alam & Asim, 2019; Wan & Duffy, 2022). However, none of these studies examined these relationships across different generations. As a result, the significance of these attributes as the main predictors of TI among the young workforce has remained unclear. ~~Prior studies mainly focused on the direct influence of PC, PP, and AC on TI, respectively. Only a few studies justified PC, PP, and AC as predictors of TI. Besides that, the relationships of JA and JS with TI have remained underexplored, which were addressed in the current study. These young employees are the future workforce and soon replace the current management. Retaining these young employees can minimise the costs of recruiting and training new talents.~~ Additionally, prior studies have mainly focused on the direct influence of PC, PP, and AC on TI and largely neglected the mechanism of how PC, PP and AC affect TI. Drawing upon the equity theory (Adams, 1965), which proposes that employees' satisfaction levels are contingent upon their perceptions of fairness regarding the relationship between their inputs and outputs, previous study has pointed out the mediating role of job satisfaction (JS) between the relationship between employee perceptions of organizational justice and employee outcomes (Mashi, 2018). Hence, the present study seeks to investigate the mechanism of how PC, PP and AC affect TI by focusing on JS.

Furthermore, limited research has explored whether there are certain conditions under which the effects of PC, PP and AC on job satisfaction and turnover intention most likely emerges. Examining these unexplored boundary conditions is critical, as it may offer valuable insights that the effects of PC, PP, and AC should not be taken for granted. According to Fried and Ferris (1987), when employees perceive more autonomous opportunities provided by the organization, they will have internal positive feelings toward their work. Job autonomy (JA) might be an important contextual resource to influence employees’ attitudes and behaviours (Morgeson & Humphrey, 2006). Hence, this study explores the role of JA within the organization as a boundary condition to the relationships of PC, PP and AC with job satisfaction and turnover intention. Overall, to address these gaps in the literature, the current study seeks to investigate whether PC, PP, and AC indirectly affect turnover intention by influencing job satisfaction under different levels of job autonomy.

~~Focusing on the young workforce in China, the current study quantitatively assessed the influence of PC, PP, and AC on JS and TI, as well as the moderating effect of JA. In particular, this study addressed the following research questions: (a) Does PC influence TI directly or indirectly under different levels of JA? (b) Does PP influence TI directly or indirectly under different levels of JA? And (c) Does AC influence TI directly or indirectly under different levels of JA?~~

**Comment 2:** The theoretical basis of the moderating effect hypothesis is not well presented. The advancement of hypothesis needs to be based on theoretical basis. The hypothesis of the moderate effect needs to be further deepened. Therefore, I propose to rearrange hypothesis 8, 9, and 10

**Author(s) Reply:** Thank you for your constructive comments and suggestions, Prof. Amendment highlighted (added – BLUE; removed RED) below:

***Job Autonomy***

Spector (1986) described JA as the extent to which employees can make their own decisions based on their judgment and preferences on how they execute the assigned tasks. Specifically, when employees have autonomy at work, they can complete tasks in a free manner according to their judgment and preferences. The job characteristics model (Hackman & Oldham, 1975) proposed that core job characteristics including autonomy generate a sense of responsibility for outcomes, meaningfulness, and knowledge of results, which in turn elicits intrinsic work motivation and job satisfaction. Autonomous jobs are expected to encourage higher levels of job satisfaction than controlled jobs because JA makes employees feel self-determined and free from external controls or constraints (Naqvi et al., 2013). As a job resource, JA can contribute to positive work outcomes (Schaufeli & Taris, 2014). Consistent with the studies above, Morgeson and Humphrey (2006) have pointed out that JA is an important contextual resource to positively influence employees’ attitudes and behaviours. Thus, the current study expects a boosting effect of job autonomy on job satisfaction.

Previous studies have mainly examined the moderating role of job resources on the relationship between job demands and work outcomes (Xanthopoulou et al., 2007; Tadić et al., 2015; Shantz & Alfes, 2015; Mayende & Musenze, 2018). Hakanen et al. (2005) also suggested that resources like job autonomy could moderate the impact of job demands for the majority of jobs. Prior researches have endeavoured to elucidate the degree to which job resources, such as job autonomy, act as a buffer in the association between variables, namely the relationship between job demands and work outcomes. Nevertheless, it is noteworthy that scant studies have explored the moderating effect of job resources in relationships between positive variables. Dominguez et al. (2020) have identified a need for additional research on the moderating influence of job resources in the relationships among positive variables. In response to this call, Wan and Duffy (2022) have investigated the moderating effect of JA on the association between decent work and job satisfaction, discovering that JA can strengthen the positive link between these two variables. According to Dominguez et al. (2020), promoting elements of intrinsic motivation can enhance the relationship between intrinsic motivational resources and positive work attitudes, including work engagement and job satisfaction. Therefore, it is rational to infer that JA, as an intrinsic motivational organizational resource, may boost the effects of other job resources such as PC, PP and AC on job satisfaction. Additionally, this boost effect may stem from the accumulation of job resources, as proposed by Hobfoll (2011), wherein workers who have greater autonomy experience a stronger motivational effect from job resources compared to those with lower autonomy levels. In high-autonomy jobs, young individuals who have greater decision-making power and place a premium on career development and performance-based pay are given greater freedom to demonstrate their abilities and receive corresponding incentives. This, in turn, leads to increased job satisfaction. Moreover, employees who have high levels of job autonomy feel a stronger sense of connection to their organization when they are allowed to perform their duties in a way that aligns with their personal values and goals. This sense of connection may ultimately result in higher job satisfaction. ~~The younger workforce embraces independence at the workplace and evaluates a job based on the salary, opportunities for autonomy, and the use of their talents at work (Lee et al., 2017). Employees would be intrinsically positive about their jobs when they are provided with more opportunities for autonomy (Fried & Ferris, 1987). Hackman (1980) identified JA as a highly desirable attribute of a job and noted its close relationships with job motivation and job attitudes. Li et al. (2021) postulated the direct relationship between JA and career self-management in regards to employees’ career growth and success. Wan and Duffy (2022) demonstrated the motivating effect of JA on employees and the positive influence of JA on JS. Apart from its motivating effect on JS directly, JA reflects an environment that is not restricted by formal procedures (Meyer et al., 2010). According to Chang et al. (2015), employees who perceive that their supervisors support autonomy tend to be more satisfied with their jobs and affectively committed to their organisation. Empowering leadership, which offers autonomy and developmental support, potentially contributes favourable influence on employees’ AC (Kim & Beehr, 2020). Haar and Spell (2009) highlighted the influence of different levels of JA on the perceived level of distributive fairness and its relationship to JS. Therefore, it is plausible that different levels of JA influence the influence of PC, PP, and AC on JS. As JA influences employees’ work attitudes, it serves as a crucial boundary condition for the relationships of PP, PC, and AC with JS.~~ Thus, the current study proposed the following hypotheses for testing:

***H8:*** *JA positively moderates the relationship between PC and JS.*

***H9:*** *JA positively moderates the relationship between PP and JS.*

***H10:*** *JA positively moderates the relationship between AC and JS.*

**Comment 3:** H8, H9, and H10 were rejected. The author needs to explain in detail in the conclusion of the manuscript why the author chose this moderate variable and whether the moderating effect is not valid can be supported by other literature. Otherwise, the selection of this variable would be meaningless.

**Author(s) Reply:** Thank you for your constructive comments and suggestions, Prof. Amendment highlighted (added – BLUE; removed RED) below:

Dominguez et al. (2020) have highlighted the necessity for additional research on the moderating impact of job resources in the relationships between positive variables. While previous studies have primarily concentrated on the buffering effects of job resources on job demands, little attention has been given to the moderating role of job resources. Consequently, there is an urgent need for more extensive research to gain a more comprehensive understanding of this complex interplay. Responding to this call, besides that, this study empirically proved the insignificant moderating effect of JA on the relationships of perceived career development opportunities (H_8_), perceived pay for performance (H_9_), and affective organizational commitment (H_10_) with job satisfaction. In other words, young employees take into account the importance of JA at the workplace, but different levels of job autonomy do not affect the influence of these factors on job satisfaction. This finding contrasted with previous studies, such as Iplik (2014), which showed that job autonomy moderates the relationship between employees' perceptions and job satisfaction. Similarly, Wan and Duffy (2022) found that job autonomy moderated the relationship between job resources, such as decent work, and job satisfaction. It is possible that different demographic groups could yield different results, and cultural or contextual differences may explain the current findings. Cultural differences can influence how individuals perceive and respond to job resources, such as job autonomy, which could affect the moderating role of job autonomy in the relationship between other job resources and job satisfaction. In contrast to countries that prioritize individualism, Chinese culture emphasizes group harmony and collectivism, which could lead to greater acceptance of limited job autonomy and a focus on fulfilling assigned responsibilities (Hong et al., 2018). Therefore, future research is encouraged to explore the moderating effect of job autonomy between positive variables in diverse contexts.

**REVIEWER #2:**

Dear Author
Thank you for sending your paper "Career Development Opportunity, Remuneration, and Organisational Commitment on Job Satisfaction and Turnover Intention: Moderating Effect of Job Autonomy" to this journal "PLOS One". I read your paper and gave my concern down here:

**Comment 4:** I suppose that the topic is too long and can be shorten.

**Author(s) Reply:** Thank you for your constructive comments and suggestions, Prof. Amendment highlighted (added – BLUE; removed RED) below:

**INTRODUCTION**

~~It has become increasingly crucial for companies to enhance their work efficiency due to the intense market competition. The workforce has substantial influence on the organisational or business success, which explains the significant need for companies to optimise their workforce, particularly the younger workforce considering their high turnover rate and independent nature (Ramlah et al., 2021).~~ With the constant development of the economy and the increasing competition in the labour market, an increasing number of young employees are contemplating job-hopping or leaving their current company. The higher the turnover intention, the higher the likelihood of quitting the job (Jacobs & Roodt, 2008). Belete (2018) described turnover intention as the intention to leave the workplace. The management must have good understanding of the factors that contribute to turnover intention considering how a higher turnover rate would affect the organisational morale, reduce the sense of identity within the company (Joe et al., 2018), and yield negative financial impact since the companies have to allocate resources for recruitment and training and experience brain drain and lower productivity (Belete, 2018).

The older workforce possesses more traditional values, such as contributing to the country and supporting the family, but the younger workforce emphasises more worldly values and embraces self-development as the key purpose of working (Zhao, 2018). In general, the younger workforce demonstrates unique characteristics at work. They have different expectations at work and expect higher expectations on perceived career development opportunity (PC) and perceived pay for performance (PP) than the older workforce (Magni & Manzoni, 2020). Besides that, prior studies demonstrated lower affective organisational commitment (AC) among young employees than older employees (Singh & Gupta, 2015; Salminen & Miettinen, 2019). ~~Adding to that, the younger workforce highly values job autonomy (JA) at the workplace. Morgeson and Humphrey (2006) defined JA as the extent of freedom and independence allowed for employees to arrange their work schedule, make decisions, and select the method of performing tasks at the workplace. The younger workforce is more independent in nature (Ramlah et al., 2021) and may be less loyal towards the company (Singh & Gupta, 2015). They are not willing to obey orders and instructions, as they prefer to work freely and independently (Lee et al., 2017). Moreover, the younger workforce emphasises how they feel, which makes them highly value job satisfaction (JS) at the workplace (Yi et al., 2010; Zhao, 2018).~~ In short, unlike the older workforce, the younger workforce has higher expectations on PC and PP but lower expectations on AC compared with older workforce ~~and display higher concern for JA and JS.~~

Prior studies examined the influence of these unique attributes of young workforce on turnover intention (TI) (Lee et al., 2017; Belete, 2018; Alam & Asim, 2019; Wan & Duffy, 2022). However, none of these studies examined these relationships across different generations. As a result, the significance of these attributes as the main predictors of TI among the young workforce has remained unclear. ~~Prior studies mainly focused on the direct influence of PC, PP, and AC on TI, respectively. Only a few studies justified PC, PP, and AC as predictors of TI. Besides that, the relationships of JA and JS with TI have remained underexplored, which were addressed in the current study. These young employees are the future workforce and soon replace the current management. Retaining these young employees can minimise the costs of recruiting and training new talents.~~ Additionally, prior studies have mainly focused on the direct influence of PC, PP, and AC on TI and largely neglected the mechanism of how PC, PP and AC affect TI. Drawing upon the equity theory (Adams, 1965), which proposes that employees' satisfaction levels are contingent upon their perceptions of fairness regarding the relationship between their inputs and outputs, previous study has pointed out the mediating role of job satisfaction (JS) between the relationship between employee perceptions of organizational justice and employee outcomes (Mashi, 2018). Hence, the present study seeks to investigate the mechanism of how PC, PP and AC affect TI by focusing on JS.

Furthermore, limited research has explored whether there are certain conditions under which the effects of PC, PP and AC on job satisfaction and turnover intention most likely emerges. Examining these unexplored boundary conditions is critical, as it may offer valuable insights that the effects of PC, PP, and AC should not be taken for granted. According to Fried and Ferris (1987), when employees perceive more autonomous opportunities provided by the organization, they will have internal positive feelings toward their work. Job autonomy (JA) might be an important contextual resource to influence employees’ attitudes and behaviours (Morgeson & Humphrey, 2006). Hence, this study explores the role of JA within the organization as a boundary condition to the relationships of PC, PP and AC with job satisfaction and turnover intention. Overall, to address these gaps in the literature, the current study seeks to investigate whether PC, PP, and AC indirectly affect turnover intention by influencing job satisfaction under different levels of job autonomy.

~~Focusing on the young workforce in China, the current study quantitatively assessed the influence of PC, PP, and AC on JS and TI, as well as the moderating effect of JA. In particular, this study addressed the following research questions: (a) Does PC influence TI directly or indirectly under different levels of JA? (b) Does PP influence TI directly or indirectly under different levels of JA? And (c) Does AC influence TI directly or indirectly under different levels of JA?~~

**Comment 5:** The research problem can be made crystal clear. Moreover, the very first sentence of the introduction section started with "it"! What does it mean? 'It' should be clear.

**Author(s) Reply:** Thank you for your constructive comments and suggestions, Prof. Amendment highlighted (added – BLUE; removed RED) below:

**INTRODUCTION**

~~It has become increasingly crucial for companies to enhance their work efficiency due to the intense market competition. The workforce has substantial influence on the organisational or business success, which explains the significant need for companies to optimise their workforce, particularly the younger workforce considering their high turnover rate and independent nature (Ramlah et al., 2021).~~ With the constant development of the economy and the increasing competition in the labour market, an increasing number of young employees are contemplating job-hopping or leaving their current company. The higher the turnover intention, the higher the likelihood of quitting the job (Jacobs & Roodt, 2008). Belete (2018) described turnover intention as the intention to leave the workplace. The management must have good understanding of the factors that contribute to turnover intention considering how a higher turnover rate would affect the organisational morale, reduce the sense of identity within the company (Joe et al., 2018), and yield negative financial impact since the companies have to allocate resources for recruitment and training and experience brain drain and lower productivity (Belete, 2018).

…….

Prior studies examined the influence of these unique attributes of young workforce on turnover intention (TI) (Lee et al., 2017; Belete, 2018; Alam & Asim, 2019; Wan & Duffy, 2022). However, none of these studies examined these relationships across different generations. As a result, the significance of these attributes as the main predictors of TI among the young workforce has remained unclear. ~~Prior studies mainly focused on the direct influence of PC, PP, and AC on TI, respectively. Only a few studies justified PC, PP, and AC as predictors of TI. Besides that, the relationships of JA and JS with TI have remained underexplored, which were addressed in the current study. These young employees are the future workforce and soon replace the current management. Retaining these young employees can minimise the costs of recruiting and training new talents.~~ Additionally, prior studies have mainly focused on the direct influence of PC, PP, and AC on TI and largely neglected the mechanism of how PC, PP and AC affect TI. Drawing upon the equity theory (Adams, 1965), which proposes that employees' satisfaction levels are contingent upon their perceptions of fairness regarding the relationship between their inputs and outputs, previous study has pointed out the mediating role of job satisfaction (JS) between the relationship between employee perceptions of organizational justice and employee outcomes (Mashi, 2018). Hence, the present study seeks to investigate the mechanism of how PC, PP and AC affect TI by focusing on JS.

Furthermore, limited research has explored whether there are certain conditions under which the effects of PC, PP and AC on job satisfaction and turnover intention most likely emerges. Examining these unexplored boundary conditions is critical, as it may offer valuable insights that the effects of PC, PP, and AC should not be taken for granted. According to Fried and Ferris (1987), when employees perceive more autonomous opportunities provided by the organization, they will have internal positive feelings toward their work. Job autonomy (JA) might be an important contextual resource to influence employees’ attitudes and behaviours (Morgeson & Humphrey, 2006). Hence, this study explores the role of JA within the organization as a boundary condition to the relationships of PC, PP and AC with job satisfaction and turnover intention. Overall, to address these gaps in the literature, the current study seeks to investigate whether PC, PP, and AC indirectly affect turnover intention by influencing job satisfaction under different levels of job autonomy.

~~Focusing on the young workforce in China, the current study quantitatively assessed the influence of PC, PP, and AC on JS and TI, as well as the moderating effect of JA. In particular, this study addressed the following research questions: (a) Does PC influence TI directly or indirectly under different levels of JA? (b) Does PP influence TI directly or indirectly under different levels of JA? And (c) Does AC influence TI directly or indirectly under different levels of JA?~~

**Comment 6:** The hypothesis on moderating effect needs further improvement.

**Author(s) Reply:** Thank you for your constructive comments and suggestions, Prof. Amendment highlighted (added – BLUE; removed RED) below:

***Job Autonomy***

Spector (1986) described JA as the extent to which employees can make their own decisions based on their judgment and preferences on how they execute the assigned tasks. Specifically, when employees have autonomy at work, they can complete tasks in a free manner according to their judgment and preferences. The job characteristics model (Hackman & Oldham, 1975) proposed that core job characteristics including autonomy generate a sense of responsibility for outcomes, meaningfulness, and knowledge of results, which in turn elicits intrinsic work motivation and job satisfaction. Autonomous jobs are expected to encourage higher levels of job satisfaction than controlled jobs because JA makes employees feel self-determined and free from external controls or constraints (Naqvi et al., 2013). As a job resource, JA can contribute to positive work outcomes (Schaufeli & Taris, 2014). Consistent with the studies above, Morgeson and Humphrey (2006) have pointed out that JA is an important contextual resource to positively influence employees’ attitudes and behaviours. Thus, the current study expects a boosting effect of job autonomy on job satisfaction.

Previous studies have mainly examined the moderating role of job resources on the relationship between job demands and work outcomes (Xanthopoulou et al., 2007; Tadić et al., 2015; Shantz & Alfes, 2015; Mayende & Musenze, 2018). Hakanen et al. (2005) also suggested that resources like job autonomy could moderate the impact of job demands for the majority of jobs. Prior researches have endeavoured to elucidate the degree to which job resources, such as job autonomy, act as a buffer in the association between variables, namely the relationship between job demands and work outcomes. Nevertheless, it is noteworthy that scant studies have explored the moderating effect of job resources in relationships between positive variables. Dominguez et al. (2020) have identified a need for additional research on the moderating influence of job resources in the relationships among positive variables. In response to this call, Wan and Duffy (2022) have investigated the moderating effect of JA on the association between decent work and job satisfaction, discovering that JA can strengthen the positive link between these two variables. According to Dominguez et al. (2020), promoting elements of intrinsic motivation can enhance the relationship between intrinsic motivational resources and positive work attitudes, including work engagement and job satisfaction. Therefore, it is rational to infer that JA, as an intrinsic motivational organizational resource, may boost the effects of other job resources such as PC, PP and AC on job satisfaction. Additionally, this boost effect may stem from the accumulation of job resources, as proposed by Hobfoll (2011), wherein workers who have greater autonomy experience a stronger motivational effect from job resources compared to those with lower autonomy levels. In high-autonomy jobs, young individuals who have greater decision-making power and place a premium on career development and performance-based pay are given greater freedom to demonstrate their abilities and receive corresponding incentives. This, in turn, leads to increased job satisfaction. Moreover, employees who have high levels of job autonomy feel a stronger sense of connection to their organization when they are allowed to perform their duties in a way that aligns with their personal values and goals. This sense of connection may ultimately result in higher job satisfaction. ~~The younger workforce embraces independence at the workplace and evaluates a job based on the salary, opportunities for autonomy, and the use of their talents at work (Lee et al., 2017). Employees would be intrinsically positive about their jobs when they are provided with more opportunities for autonomy (Fried & Ferris, 1987). Hackman (1980) identified JA as a highly desirable attribute of a job and noted its close relationships with job motivation and job attitudes. Li et al. (2021) postulated the direct relationship between JA and career self-management in regards to employees’ career growth and success. Wan and Duffy (2022) demonstrated the motivating effect of JA on employees and the positive influence of JA on JS. Apart from its motivating effect on JS directly, JA reflects an environment that is not restricted by formal procedures (Meyer et al., 2010). According to Chang et al. (2015), employees who perceive that their supervisors support autonomy tend to be more satisfied with their jobs and affectively committed to their organisation. Empowering leadership, which offers autonomy and developmental support, potentially contributes favourable influence on employees’ AC (Kim & Beehr, 2020). Haar and Spell (2009) highlighted the influence of different levels of JA on the perceived level of distributive fairness and its relationship to JS. Therefore, it is plausible that different levels of JA influence the influence of PC, PP, and AC on JS. As JA influences employees’ work attitudes, it serves as a crucial boundary condition for the relationships of PP, PC, and AC with JS.~~ Thus, the current study proposed the following hypotheses for testing:

***H8:*** *JA positively moderates the relationship between PC and JS.*

***H9:*** *JA positively moderates the relationship between PP and JS.*

***H10:*** *JA positively moderates the relationship between AC and JS.*

**Comment 7:** The methodology section seems alright.

**Author(s) Reply:** Thank you for your constructive comments and suggestions, Prof. Amendment highlighted (added – BLUE; removed RED) below:

**METHODOLOGY**

**Data Collection**

The young workforce in China generally has lower organisational loyalty and is associated with higher turnover rate (Warner & Zhu, 2018; Fang et al., 2020). Therefore, it was deemed significant for the current study to assess the influence of PC, PP, and AC on JS and TI, as well as the moderating effect of JA among the young employees in China. Referring to the China State Council (2022), youths are of those between 14 and 35 years old. Considering the context of the current study, working individuals of between 18 and 35 years old in China were targeted.

This research employed a quantitative approach to examine the relationship between variables. G*Power 3.1 was used to calculate the minimum sample size required to achieve the target analysis level and the desired minimum sample size with a power of 0.95 and an effect size of 0.15, taking into account the five predictors in this study. The output result indicates that at least 138 valid samples are required (Faul et al., 2007). However, for partial least squares-structural equation modeling analysis, it is recommended to use a minimum of 200 samples (Hair, 2017). Online data collection was conducted between July 1, 2022 and August 8, 2022. The online survey was administered through WJX (<http://www.wjx.cn/>), a functional and user-friendly tool. In order to ensure that all participants were young Chinese adults who were employed and aligned with the background of this study, judgmental questions were used for exclusion purposes. Prior to the commencement of the formal questionnaire, all participants signed an informed consent form, in which they were informed of the purpose of the data collection, the final destination of the data, and their right to withdraw from participation at any time. ~~Using the G-Power 3.1, this study estimated the minimum sample size required. With the power of 0.95, effect size of 0.15, and six predictors, this study was expected to gather at least 146 effective samples (Faul et al., 2007). According to Hair (2017), a sample size of at least 200 is recommended for partial least squares-structural equation modelling (PLS-SEM). Therefore, this study opted for a sample size of at least 500 respondents to reduce the effects of low sample size. Convenience sampling strategy was employed in this study. An online survey was conducted via WJX (~~[~~http://www.wjx.cn/~~](http://www.wjx.cn/)~~), which is a functional and user-friendly tool. The online survey included several qualifying questions to filter potential respondents.~~ At the end of the data collection, this study successfully obtained 532 valid questionnaire sets.

**Comment 8:** I missed the gravity of the discussion section in this manuscript. Would you add discussion section here?

**Author(s) Reply:** Thank you for your constructive comments and suggestions, Prof. Amendment highlighted (added – BLUE; removed RED) below:

**DISCUSSION AND IMPLICATIONS**

This cross-sectional study exclusively focused on turnover intention of young employees in China. Retaining young employees is undoubtedly a major challenge for organisations today. Therefore, it is crucial for organisations to understand how attached their employees are to the workplace. Focusing on that, the current study presented empirical evidence on factors that influence TI. Firstly, this study empirically proved the direct significant influence of perceived career development opportunities on job satisfaction (H_1_) and turnover intention (H_2_). Stahl et al. (2009) and Ohunakin et al. (2018) presented similar findings on the substantial influence of perceived career development opportunities on turnover intention. Likewise, Price and Reichert (2017) identified perceived career development opportunities as one of the factors that can enhance job satisfaction. In other words, PC significantly influences JS and TI. This study also empirically demonstrated the significant and positive influence of perceived pay for performance on job satisfaction (H_3_). This finding was consistent with the studies conducted by Ren et al. (2017) and Bae (2021), which have reported the significant and positive relationship between perceived pay for performance and job satisfaction. This finding highlighted the importance of perceived pay for performance in motivating young Chinese employees and improving job satisfaction. Additionally, the study discovered that perceived pay for performance had a negative impact on turnover intention, providing support for H_4_. This finding aligned with the assertions by Kuvaas et al. (2016) and Hur and Ha (2019). They pointed out performance-based pay had a negative impact on turnover intention. In other words, among young Chinese employees, the recognition and appropriate compensation of their efforts and contributions may lead to a decreased likelihood of contemplating job departure~~., which were found to be only partially consistent with the results reported by Ren et al. (2017). The prior study reported the significant and positive relationship between PP and JS but insignificant relationship between PP and TI. Nonetheless, the current study hypothesised the significant and negative influence of PP on TI. Based on the results, H4 was supported.~~

Besides that, this study empirically demonstrated the positive influence of affective organizational commitment on job satisfaction. Thus H_5_ was supported. This finding was consistent with several prior studies (Ozturk et al., 2014; Yang et al., 2019; Cao et al., 2020). In other words, employees who have a strong emotional attachment to their organization are more likely to experience job satisfaction. Furthermore, the current study shed light on the relationship between affective organizational commitment and turnover intention in the context of young Chinese workers. Specifically, the study revealed that employees' affective organizational commitment negatively influenced their turnover intentions (H_6_). Gara Bach Ouerdian et al. (2021) and Karatepe (2022) presented similar findings on the substantial influence of affective organizational commitment on turnover intention. The current study adds to the existing literature on the importance of job resources such as affective organizational commitment in reducing employee turnover intention, which has implications for organizations seeking to improve their retention rate. The obtained results of this study also confirmed JS as a significant determinant of TI (H_7_), which supported the findings of several prior studies (Alam & Asim, 2019; Wan & Duffy, 2022). In other words, the significance of JS as a predictor of TI should not be overlooked.

~~Besides that, this study empirically demonstrated the positive influence of AC on JS (H5) and the negative influence of AC on TI (H6), which supported the findings of several prior studies (Yang et al., 2019; Cao et al., 2020; Obeng et al., 2021). In other words, young employees with AC display lower TI and tend to express higher JS. However, there have varied findings on the direction of the relationship between AC and JS. For instance, Chordiya et al. (2017) identified JS as the most important antecedent of AC, while Berta et al. (2018) highlighted ambiguous direction of the positive relationship between AC and JS.~~

Dominguez et al. (2020) have highlighted the necessity for additional research on the moderating impact of job resources in the relationships between positive variables. While previous studies have primarily concentrated on the buffering effects of job resources on job demands, little attention has been given to the moderating role of job resources. Consequently, there is an urgent need for more extensive research to gain a more comprehensive understanding of this complex interplay. Responding to this call, Besides that, this study empirically proved the insignificant moderating effect of JA on the relationships of perceived career development opportunities (H_8_), perceived pay for performance (H_9_), and affective organizational commitment (H_10_) with job satisfaction. In other words, young employees take into account the importance of JA at the workplace, but different levels of job autonomy do not affect the influence of these factors on job satisfaction. This finding contrasted with previous studies, such as Iplik (2014), which showed that job autonomy moderates the relationship between employees' perceptions and job satisfaction. Similarly, Wan and Duffy (2022) found that job autonomy moderated the relationship between job resources, such as decent work, and job satisfaction. It is possible that different demographic groups could yield different results, and cultural or contextual differences may explain the current findings. Cultural differences can influence how individuals perceive and respond to job resources, such as job autonomy, which could affect the moderating role of job autonomy in the relationship between other job resources and job satisfaction. In contrast to countries that prioritize individualism, Chinese culture emphasizes group harmony and collectivism, which could lead to greater acceptance of limited job autonomy and a focus on fulfilling assigned responsibilities (Hong et al., 2018). Therefore, future research is encouraged to explore the moderating effect of job autonomy between positive variables in diverse contexts.

**Theoretical Implications**

This paper supports the call for research into the impact of employees' interpretations of HRM practices on the effectiveness of the HRM system (Kehoe & Wright, 2013; Ren et al., 2017). This study presented significant theoretical implications that would be of much interest for future research on TI, particularly among young employees. This study addressed the call of prior studies (Brokmeier et al., 2022) on the need for more in-depth insights on how job characteristics influence the formation of work attitudes among employees. This study presented empirical evidence on factors that can reduce young employees’ TI in relation to the unique attributes of this young workforce. In particular, the significance of PC, PP, and AC in influencing TI was highlighted. This study demonstrated the importance of considering the work characteristics of young employees when it comes to exploring the formation of their JS and TI. This study also presented unexpected findings on how JA does not moderate the relationships of PC, PP, and AC with JS despite the importance of JA for young employees at the workplace.

**Practical Implications**

~~This study also presented several practical implications that would be of much interest for various organisations and their management. First, this study clearly demonstrated the importance of enhancing PC, PP, and AC for employees to have higher JS and subsequently, TI.~~ The results of this study hold significant practical implications for organizations, particularly those operating in China, seeking to reduce turnover intention among young employees. To attain this objective, organizations must concentrate on augmenting perceived career development opportunities, perceived pay for performance, and affective organizational commitment. To improve employee perceptions of career development opportunities, organizations can facilitate training and development programs designed to enhance employees' skills and competencies, provide opportunities for job rotation, and mentorship programs. Furthermore, managers can implement performance-based pay systems, furnish regular feedback and recognition to employees, and cultivate a culture of accountability and transparency. Such measures will elevate employees' perception of fairness in the pay structure and stimulate their motivation to perform well.

This study also demonstrated AC as the most important element with the highest overall effects on TI among young employees in China. However, it is the least-valued element by the management. Therefore, organizations should prioritize the development of strategies and initiatives that enhance employees' affective commitment to the organization. Organizations can create a positive work environment that fosters trust, openness, and communication (Men & Yue, 2019). This can be achieved by promoting a strong organizational culture, providing opportunities for employee involvement and participation in decision-making, and recognizing and rewarding employees' contributions. ~~This study prompted the crucial need for the management to identify strategic ways that can enhance PP, PC, and AC in order to reduce TI among young employees.~~

Wish you all the best.

6. PLOS authors have the option to publish the peer review history of their article ([what does this mean?](https://journals.plos.org/plosone/s/editorial-and-peer-review-process#loc-peer-review-history)). If published, this will include your full peer review and any attached files.

If you choose “no”, your identity will remain anonymous but your review may still be made public.


**Do you want your identity to be public for this peer review?** For information about this choice, including consent withdrawal, please see our [Privacy Policy](https://www.plos.org/privacy-policy).

Reviewer #1: No

Reviewer #2: **Yes:** Md. Aftab Uddin
